# The Synergistic Effect of Calcained Coal-Series Kaolinite and Limestone on the Hydration of Portland Cement

**DOI:** 10.3390/ma17184512

**Published:** 2024-09-13

**Authors:** Jin Tang, Yue Yu, Yuanqing Bu, Bing Ma, Hao Zhou, Rong Zhou, Jiaqing Wang, Houhu Zhang

**Affiliations:** 1 Nanjing Institute of Environmental Sciences, Ministry of Ecology and Environment of the Peopole’s Republic of China, Nanjing 210042, China; tangjin@nies.org (J.T.); yuyue@nies.org (Y.Y.); byq@nies.org (Y.B.); myyanb@aliyun.com (B.M.); zhourong@nies.org (R.Z.); 2 College of Civil Engineering, Nanjing Forestry University, Nanjing 210037, China; jiaqingw@njfu.edu.cn

**Keywords:** calcined coal-series kaolinite, limestone, synergistic effect, carboaluminates

## Abstract

Limestone calcined clay cement (LC^3^) presents a promising alternative material due to its reduced CO_2_ emissions and superior mechanical properties compared to traditional Portland cement (PC). This study investigates the synergistic effect of calcined coal-series kaolinite (CCK) and limestone (LS) on the hydration behavior of cement, specifically focusing on varying mass ratios. The combination of CCK and LS promotes the formation of strätlingite and carboaluminates, which enhances early-age strength development. Additionally, the inclusion of CCK facilitates the formation of carboaluminates during later stages of hydration. After 56 days of hydration, the content of carboaluminates is over 10%wt. This stimulation of secondary hydration products significantly refines the evolution of pore structure, with the harmful large pores gradually transformed into harmless medium pores and gel pores, leading to marked improvements in compressive strength from 7 to 28 days. Replacing 45% PC with CCK and LS at mass ratio of 7 to 2, the compressive strength of blends reaches 47.2 MPa at 28 days. Overall, the synergistic interaction between CCK and LS presents unique opportunities to minimize the CO_2_ footprint of the cement industry without compromising early and long-term performance.

## 1. Introduction

The most effective strategy for mitigating the carbon footprint of the cement industry involves the partial substitution of clinker with supplementary cementitious materials (SCMs) [1,2]. In recent decades, the use of blended cements has increased to address the need for clinker reduction [3,4]. However, the application of traditional SCMs, such as fine limestone (LS), fly ash, and slag, is limited in their replacement levels, which restricts the potential for achieving a significant reduction in the clinker factor beyond 15% [5,6].

The synergistic effect of limestone and calcined clay contributes to the formation of additional hydration products and refines the pore structure of the cement during hydration [7]. Kaolinite-rich clays have gained increased attention for their potential to reduce the clinker factor in cement production. Compared to reference Portland cement (PC), limestone calcined clay cement (LC^3^) exhibits similar or superior mechanical properties, enhanced pore structure evolution, and improved resistance to alkali–silica reactions and chloride penetration starting from 7 days of curing, even when employing a high replacement level of 50% [8,9]. Furthermore, LC^3^ can reduce CO_2_ emissions by 30% to 40% at a consistent mass level compared to PC [10,11].

Previous studies have indicated that the kaolinite content of clay is a significant factor influencing the mechanical, hydration, and durability performance of LC^3^ [12,13,14]. However, kaolinite-rich clay also has high-value applications in industries such as ceramics, paper-making, and rubber. In contrast, clay with low kaolinite content is widely available globally [15]. Recent studies have focused on the effects of low-grade calcined clay and limestone on the hydration and mechanical properties of PC [14,16]. Key parameters investigated include the kaolinite content in the clay, the proportion of each raw material, and the cement substitution level [17,18]. Given that clay is a valuable resource in China, developing alternative clay materials as SCMs has positive implications for sustainable construction practices [19].

Coal-series kaolinite, a byproduct of coal mining, constitutes approximately 10% to 15% of annual coal production emissions, with reserves in China exceeding 4 billion tons to date. This type of kaolinite is rich in kaolinite, while impurities include quartz, carbon, and calcium carbonate, among others [20,21]. Similar to kaolinite clay, coal-series kaolinite undergoes thermal activation at temperatures between 500 °C and 900 °C, converting into metakaolin, which can serve as a sustainable pozzolan for cementitious phases [22]. During cement hydration, coal-series kaolinite reacts with portlandite to produce additional hydration products, such as calcium silicate hydrate (C-S-H), calcium aluminate hydrate (C-A-H), and calcium aluminosilicate hydrate (C-A-S-H), as represented in Equations (1) and (2).

(1) $$AS_{2}+6CH+9H\to 2C-S-H+C_{4}AH_{13}$$

(2) $$AS_{2}+3CH+6H\to C-S-H+C_{2}ASH_{8}$$

where $AS_{2}$ means metakaolin, CH means portlandite, and H indicates water.

It is well established that LS reacts with tricalcium aluminate (C_3_A) to form hemicarboaluminate (HC) and monocarboaluminate (MC), which improves the pore structure of the system during hydration [23,24]. This reaction promotes the stabilization of ettringite rather than its conversion into monosulfoaluminate. Lothenbach et al. reported that aluminum uptake in calcium silicate hydrate (C-S-H) plays a significant role in the LS–Portland cement system, indicating that the reactivity of the LS depends on the aluminum content (from C_3_A and tetracalcium aluminoferrite (C_4_AF)) and the sulfate content of the PC [25,26]. The pozzolanic reaction of CCK introduces additional aluminum into the gel during cement hydration, suggesting that a greater substitution of clinker can be achieved in the ternary system [27,28]. The synergistic effect of CCK and LS enhances the mechanical properties of the system, allowing for a reduction in clinker content by up to 50%, as illustrated in Equation (3) [29].

(3) $$A+C\bar{C}+3CH+H\to C_{3}A\cdot C\bar{C}\cdot H_{12}$$

where A indicates Al in hydrated gel, $C\bar{C}$ means calcite, $C_{3}A\cdot C\bar{C}\cdot H_{12}$ is MC.

Several studies have examined the effects of cement replacement levels, the quality of calcined clay, the replacement ratios of calcined clay, and other influencing factors on the strength development of LC^3^ [30,31]. The synergistic effect of calcined clay and limestone optimized the pore structure evolution and hydration products; the workability and durability of the LC^3^ improved [32,33].

Our previous research indicated that the incorporation of metakaolin consistently promotes the formation of HC and MC, refining the pore structure of blends during cement hydration [34]. When CCK and LS are mixed in a 2:1 ratio to substitute 30% of the cement, the mechanical properties of the sample exceed those of reference PC after 7 days of hydration [35]. Cardinard et al. reported that low-grade calcined clay exhibits a comparable degree of reactivity to that of high-grade calcined clay during long-term hydration [36]. Kaminskas et al. found that the pozzolanic reaction of calcined clay leads to the formation of strätlingite (C_2_ASH_8_), contributing to strength development at an early age [37]. The formation of HC and MC further enhances strength development at later ages. Additionally, Sun et al. discovered that the addition of calcium hydroxide (CH) effectively enhances the reaction of calcined clay, significantly improving the formation of carboaluminates [8].

Previous research has indicated that the formation of carboaluminates significantly influences pore structure and strength development [38,39]. Investigating the reaction between LS and its relationship with pore structure evolution and strength development is essential for understanding the hydration mechanisms of LC^3^. In this study, we manipulate and control the mass ratio of CCK and LS to develop novel LC^3^ with enhanced properties.

## 2. Materials and Methods

### 2.1. Raw Materials and Mixture Design

**Figure 1:**
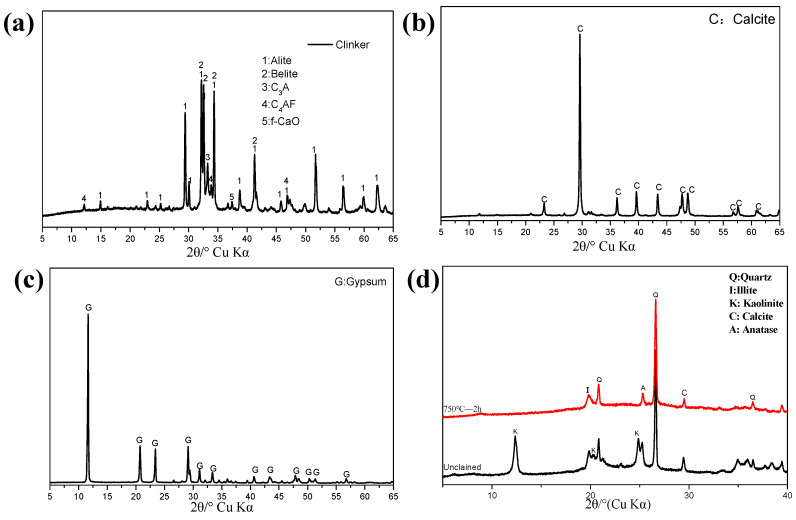
XRD patterns of (**a**) clinker, (**b**) limestone, (**c**) gypsum, and (**d**) coal-series kaolinite before and after 750 °C 2 h calcination.

**Figure 2:**
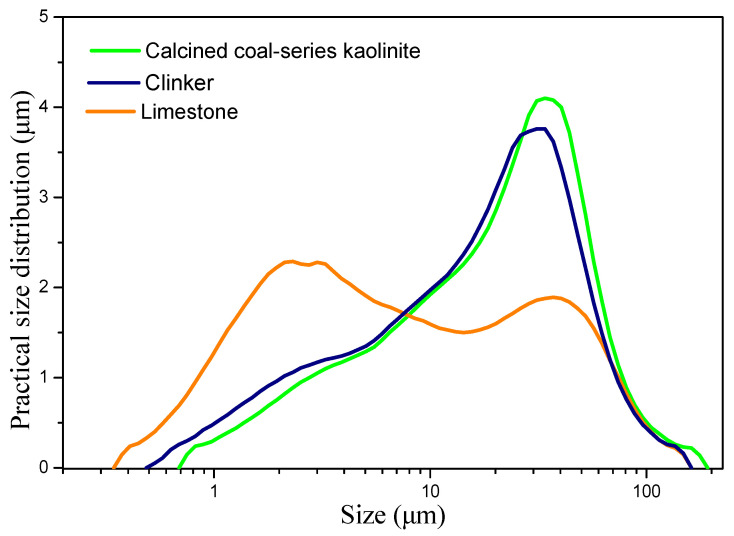
Particle size distribution curve of raw materials.

In this study, clinker, limestone, coal-series kaolinite, and gypsum were utilized to prepare cementitious materials. Clinker and gypsum were procured from Nanjing China United Cement Co., Ltd., while the LS was obtained from Suzhou Conch Cement Co., Ltd., and the coal-series kaolinite was sourced from a coal mine in Yunnan Province. The chemical composition of the raw materials, along with the mineral composition of the clinker, is presented in Table 1. The phase compositions of the raw materials were analyzed using X-ray diffraction (XRD), as illustrated in Figure 1. Prior to the experiments, the raw materials were ground, and Figure 2 depicts the particle size distribution curve of the raw materials. The D50 values for the clinker, calcined coal-series kaolinite, and limestone were 17.59 μm, 18.84 μm, and 6.17 μm, respectively.

The coal-series kaolinite was calcined at 750 °C for 2 h in a muffle furnace to remove carbon and transform the kaolinite into metakaolin. The chemical compositions and X-ray diffraction (XRD) patterns of the coal-series kaolinite before and after calcination are presented in Table 1 and Figure 1d. Utilizing Equation (4), the kaolinite content in the coal-series kaolinite was calculated to be 47.75% by evaluating the dehydroxylation process of kaolinite during thermogravimetric (TG) analysis, as outlined in Equation (5).

(4) $$AS_{2}H_{2}\to AS_{2}+2H$$

(5) $${Wt.\%}_{kaolinite}=\frac{W_{350}-W_{575}}{W_{350}}\times \frac{258}{36}\times 100\%$$

where $AS_{2}H_{2}$ indicates kaolinite. ${Wt.\%}_{kaolinite}$ means the kaolinite content of coal-series kaolinite, $W_{350}$ and $W_{575}$ mean the weight of sample at 350 °C and 575 °C during TG experiment.

The ternary cementitious system was formulated using clinker, gypsum, LS, and CCK, maintaining a constant substitution rate of 45% for the latter two components. The detailed composition of the blends is provided in Table 2. All raw materials were accurately weighed according to the mix design and were subsequently combined in a mixing tank for 24 h to produce homogeneous cementitious materials.

### 2.2. Sample Preparations

Cement pastes were prepared using a laboratory mixer. In this experiment, the water-to-cement ratio was controlled as 0.5. The weighed blends and water were added to the mixer and homogenized at 500 rpm for 2 min. The prepared pastes were then placed into plastic tubes and cured in a curing box maintained at 98% humidity and 20 °C until the time of testing. At specified time intervals, the cured samples were precisely sectioned and immersed in alcohol for 2 days to halt the hydration process. Subsequently, the slices were vacuum-dried at 40 °C for 2 days.

Standard mortars were cast in accordance with the Chinese Standard GB/T 17671-1999 based on the mixture design to evaluate strength evolution during hydration. A total of 450 g of blends, 225 g of water, and 1350 g standard sand were used as raw materials. The mortars were poured into steel molds and mechanically vibrated for 1 min. Afterward, the molds were stored at 20 °C for 1 day. Strength testing was conducted at 3, 7, and 28 days post-casting, with the mortar strength determined by calculating the mean strength of three samples. A consistent water-to-blend ratio of 0.5 was applied across all mixtures in this study.

### 2.3. Characterization Methods

The phase composition for raw materials and hydration productions were measured by X-ray diffraction (XRD) measurement. XRD (Rigaku SmartLab 3000A diffractometer) was performed at a scanning rate of 5°/min over scanning range 2θ of 5°–70°; the step size was controlled as 0.01°.

The content of hydration products and the hydration degree of clinker during the hydration process was characterized by Q-XRD approach. α-Al_2_O_3_ (NISR standard SRM-676a) was selected as the standard material [40]. The degree of hydration (DoH) was determined by comparing the contents of clinker’s primary minerals (C_3_S, C_2_S, C_3_A, and C_4_AF) before and after the hydration process, as shown in Equation (6).

(6) $$DoH=1-\frac{W_{C_{3}s}\left(t\right)+W_{C_{2}s}\left(t\right){+W}_{C_{3}A}\left(t\right)+W_{C_{4}AF}\left(t\right)}{W_{C_{3}s}\left(t_{0}\right)+W_{C_{2}s}\left(t_{0}\right){+W}_{C_{3}A}\left(t_{0}\right)+W_{C_{4}AF}\left(t_{0}\right)}$$

where $W_{C_{3}s}\left(t_{0}\right)$, $W_{C_{2}s}\left(t_{0}\right)$, $W_{C_{3}A}\left(t_{0}\right)$, and $W_{C_{4}AF}\left(t_{0}\right)$ represent the initial content of mineral phases in anhydrous blends before hydration, and $W_{C_{3}s}\left(t\right)$, $W_{C_{2}s}\left(t\right)$, $W_{C_{3}A}\left(t\right)$ and $W_{C_{4}AF}\left(t\right)$ represent the content of minerals in sample hydrated at t.

The heat release during early hydration at 20 °C was measured in an isothermal calorimeter (TAM Air). The cement pastes with water-to-cement ratio of 0.5 were mixed with a laboratory mixer at 500 rpm for 2 min. Then, 6 g of paste was placed in a plastic ampoule, sealed, and put in the calorimetry for 3 d.

Thermogravimetric analysis (TGA) was employed to determine the amount of bound water and CO_2_ released while heating. This measurement condition of TG (Mettler Toledo TGA/DSC1) was as follows: The heating rate, the test temperature rate, and the atmosphere are 10 ℃/min, 30 ℃ to 1000 ℃, and nitrogen gas at 30 mL/min. The formation hydration products of the cement pastes were analyzed by monitoring the mass change of sample during heating process. The bound water content of the blends was obtained by calculating mass changes between 50 °C to 400 °C, which can be used to characterize the amount of hydration products at different hydration age. Typically, the content of CH in hydrated sample was evaluated based on the weight loss range from 400 °C to 470 °C, with the anhydrous sample mass at 550 °C as reference, according to Equation (7).

(7) $${CH}_{dry}=\frac{W_{400}-W_{470}}{W_{550}}\times \frac{74}{18}$$

where $W_{400}$, $W_{470}$, and $W_{550}$ indicate the mass of sample at 400 ℃, 470 ℃, and 550 ℃ during thermal analysis.

The pore structure evolution of the samples during the hydration was characterized using Mercury intrusion porosimeter (MIP) method (Quanta chrome PoreMaster 60 GT), which can generate pressure in the range of 1.5 KPa to 350 KPa. Approximately 1 g of prepared sample (3 to 4 pieces) was used in this test.

## 3. Results and Discussion

### 3.1. Effect of CCK and LS on the Mechanical Properties

**Figure 3:**
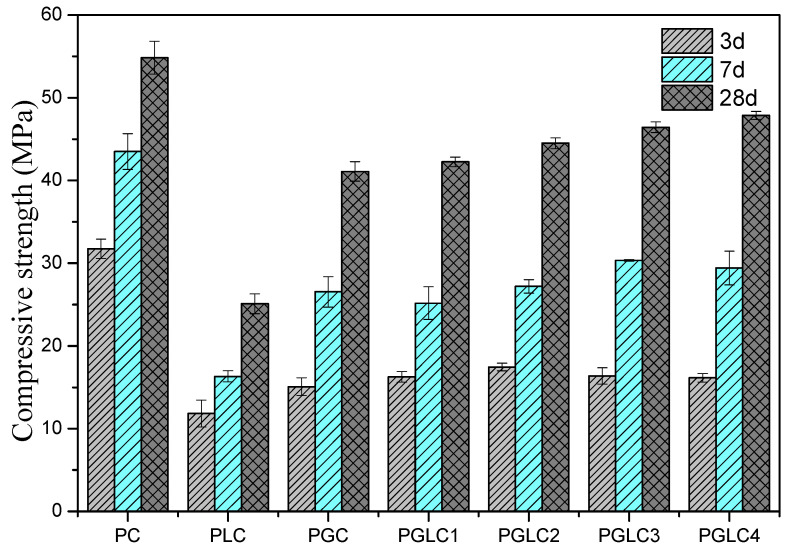
Effect of CCK and LS on the compressive strength of cement.

Figure 3 illustrates the mechanical properties of cement mortars hydrated for 3, 7, and 28 days. The strength development of the PLC sample was significantly impaired by the dilution effect of LS, resulting in a reduction of compressive strength by 62.7%, 62.4%, and 54.2% at 3, 7, and 28 days, respectively, compared to the reference PC. Previous studies have indicated that LS can enhance the hydration of cement and generate additional hydration products; however, in this case, the dilution effect of LS was the predominant factor influencing the results. The compressive strength of PGC exceeded that of PLC at all hydration ages. According to Equations (1) and (2), the formation of abundant calcium–alumino–silicate hydrate (C-(A)-S-H) gel contributed to the enhanced strength development observed in PGC [41].

At early stages of hydration, the nucleation and synergistic effects of CCK and LS enhance the mechanical properties, resulting in the strength of PGLC being approximately 5 MPa higher than that of PLC at 3 days. The mass ratio of CCK to LS shows a minimal influence on strength development during the early hydration period, as all PGLC samples exhibited similar compressive strengths at 3 days. With the exception of PGLC1, the strengths of PGLC2, PGLC3, and PGLC4 were slightly higher than that of PGC, with this trend becoming more pronounced after 28 days of hydration. This outcome indicates that the synergistic effects of CCK and LS are more advantageous to the overall strength development of the system. The incorporation of CCK and LS as SCMs is an effective way to reduce the CO_2_ footprint of cement industry. Conversely, at later hydration ages, the mass ratio of CCK to LS becomes the dominant factor influencing strength. As the mass ratio of CCK to LS increased from 1:2 to 7:2, the compressive strength of PGLC rose from 42.6 MPa to 47.2 MPa after 28 days of hydration. Although the CCK content in PGLC4 is significantly higher than in PGLC2, the strength of the blend only increased by 4.2 MPa (9.6%), indicating that there is an optimal dosage range for CCK to maximize strength development.

### 3.2. Effect of CCK and LS on the Cement Hydration

**Figure 4:**
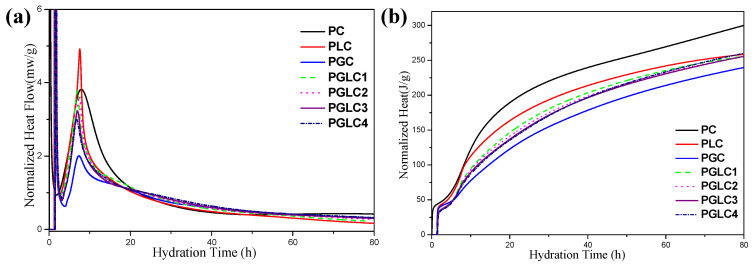
The isothermal calorimetry curves of per gram of blends (**a**) rate of hydration and (**b**) cumulative heat of hydration.

The isothermal calorimetry results for the various samples are displayed in Figure 4. Both CCK and LS accelerated the hydration of cement, particularly the hydration of tricalcium silicate (C_3_S). Notably, the peak of the acceleration period decreased with increasing CCK content, suggesting that the incorporation of CCK requires more free water than the cement itself during the mixing process.

After 1 day of hydration, the heat released by the PGLC blends exceeded that of the reference PC and PLC (see Figure 4a). This enhanced heat release can be attributed to the pozzolanic reactions of CCK and the formation of carboaluminates.

The additional hydration products contributed to the higher strength results observed at 3 days (refer to Figure 3). During the first 3 days of hydration, PLC exhibited a higher total heat release compared to the PGLC blends, indicating that the hydration of C_3_S played a dominant role in the early hydration phase.

However, after 80 h of hydration, the total heat release from PLC and PGLC blends became similar, with PGLC demonstrating a stronger heat release capacity in the later hydration stages. After 80 h of hydration, PGLC 4 blends represented 86.2% of the heat that reference PC released, it can be illustrated by the nucleation effect of SCMs promotes the hydration of C_3_S in early hydration age. This enhanced heat release is likely responsible for the further strength development of PGLC at later hydration ages.

In contrast, PGC exhibited the lowest heat release at 80 h, approximately 12 J less than the PGLC blends, after 80 h of hydration. The incorporation of CCK significantly suppressed the early hydration of C_3_S, while the dilution effect of LS optimized the early hydration process within the PC–CCK system.

**Figure 5:**
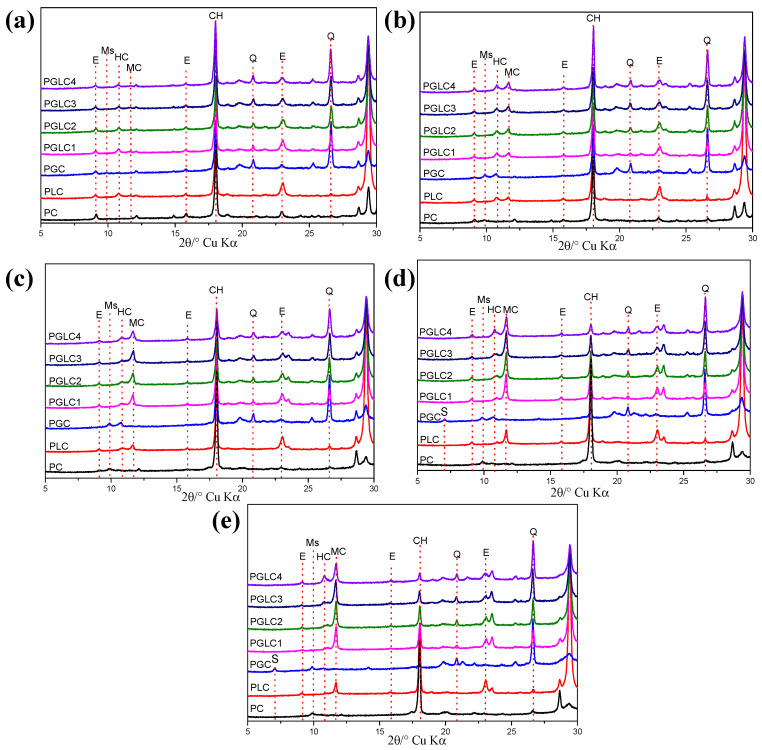
XRD patterns of blends after (**a**) 1 d, (**b**) 3 d, (**c**) 7 d, (**d**) 28 d, and (**e**) 56 d of hydration. The main peak of ettringite (E), monosulfoaluminate (Ms), hemicarboaluminate (HC), monocarboaluminate (MC), ferrite (F), portlandite (CH), strätlingite (S), and quartz (Q) were observed.

Figure 5 presents the X-ray diffraction (XRD) patterns of the blends after different hydration ages. The hydration of PC generates ettringite, which subsequently transforms into monosulfoaluminate. The presence of portlandite (CH) is maintained throughout the hydration process. As shown in Figure 5a, hydrates (HC) were observed in all blends containing LS after 1 day of hydration; the incorporation of CCK only slightly influenced the intensity of the HC diffraction peaks. This limited reaction of CCK suggests that the aluminum (Al) in the system at this stage primarily comes from the hydration of tricalcium aluminate (C_3_A).

Similar to the reference PC, the transformation from ettringite to monosulfoaluminate was observed in PGC due to the lack of LS after 3 days of hydration. The conversion of HC to MC can also be detected, with the incorporation of CCK stimulating the formation of carboaluminates; the intensity of the diffraction peaks for both HC and MC was higher than that of PLC. This trend continued at 7 days of hydration, where a more pronounced MC peak was observed alongside a decrease in the HC peak. HC persisted in PGC from 3 days onward, likely due to the carbonization of CH and LS within the CCK (refer to Figure 1d). At 28 days, the abundance of Al in CCK promoted the formation of stratlingite (C_2_ASH_8_) in PGC, which remained present even after 56 days of hydration.

However, the incorporation of LS appeared to inhibit the formation of stratlingite, as no corresponding diffraction peaks (d = 12.55, 7.05°) were found in the LS-containing mixtures. Increased Al content also stabilized the existence of HC. As the content of CCK increased, the intensity of the CH diffraction peak decreased, attributable to the pozzolanic reaction of CCK during the later stages of hydration.

**Figure 6:**
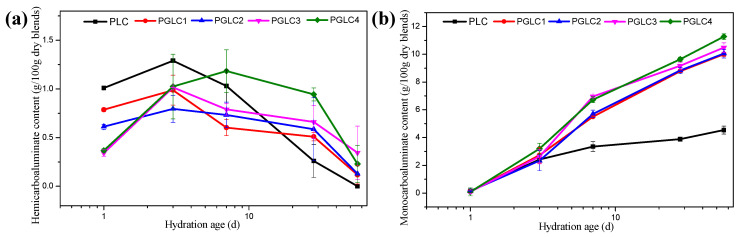
The formation of (**a**) HC and (**b**) MC during cement hydration, calculated by XRD refinement measurement.

The content of carboaluminates throughout hydration (at 1, 3, 7, 28, and 56 days) was determined using X-ray diffraction (XRD) refinement, as illustrated in Figure 6. After 1 day of hydration, the content of hydrates (HC) decreased with increasing CCK content, which can be attributed to the aluminum (Al) available at that time being primarily derived from the hydration of tricalcium aluminate (C_3_A). Thus, the incorporation of CCK had a minor effect on the early formation of HC.

Excluding the PGLC4 blend, the HC content reached its maximum at 3 days of hydration before subsequently declining. As the CCK content increased, further formation of HC was observed at 7 days, indicating that the additional CCK enhanced the reactivity of LS. The transformation of HC into monosulfoaluminate (MS) was notably evident throughout the hydration process, as the content of HC gradually decreased while the content of MC increased during the later stages of hydration.

The continued formation of MC observed in the later hydration periods suggests that the synergistic effects of CCK and LS exert a long-term influence on cement hydration. An interesting phenomenon can be observed from Figure 6b: The content of MC of all PGLC blends was over 10% after 56 days of hydration, indicating that the formation of MC is critical for the late strength development of the system. The results indicated that the CCK and LS are an excellent combination of SCMs. However, increasing the CCK content from 15% to 35%, the content of MC only increased by about 2%; increasing the content of CCK has a limited effect on the content of MC at a late hydration age.

**Figure 7:**
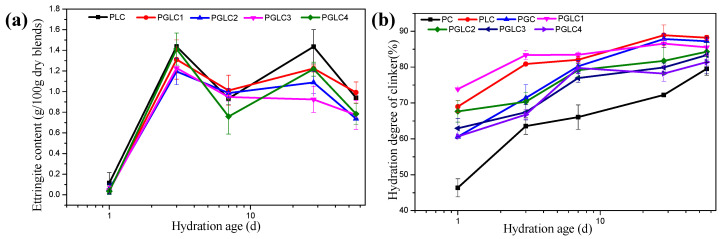
The (**a**) formation of ettringite and (**b**) DoH of clinker during cement hydration, calculated by XRD refinement measurement.

In addition to HC and MC, the content of ettringite and the degree of hydration of the clinker can be calculated through X-ray diffraction refinement, as presented in Figure 7. The incorporation of LS indirectly stabilized the presence of ettringite, with all samples exhibiting similar patterns of ettringite evolution (Figure 7a). The presence of CCK appeared to have a limited impact on the existence of ettringite, as its content was primarily influenced by the gypsum content.

Both CCK and LS enhanced the hydration of the clinker at all hydration stages, which can be attributed to the nucleation effects of SCMs. In comparison to CCK, the addition of more LS significantly improved the degree of hydration results. In correspondence with the compressive strength and carboaluminates results, the synergistic effects of CCK and LS play a dominant role in further strength development during the late stages of hydration.

**Figure 8:**
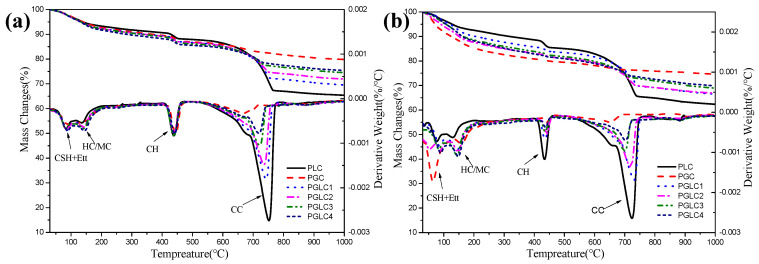
The thermogravimetric and different thermogravimetric resulted after hydrated (**a**) 3 d and (**b**) 28 d.

**Figure 9:**
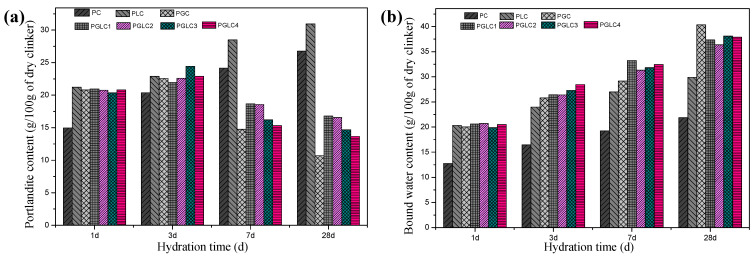
The (**a**) portlandite content and (**b**) bound water content after 1, 3, 7, and 28 d of hydration.

Figure 8 illustrates the thermogravimetric (TG) curves and the differential thermogravimetric (DTG) curves after hydration for 3 days and 28 days. Analysis of the mass changes during TG allows for the determination of the portlandite and bound water content in the blends at each hydration age, as shown in Figure 9. The incorporation of CCK stimulated the formation of carboaluminates after 3 days, with this trend further enhanced at 28 days. Additionally, as indicated in Figure 8b, the synergistic effect of CCK and LS contributes to the generation of additional C-(A)-S-H gel by consuming portlandite. This increase in hydration products leads to improved mechanical properties.

The content of portlandite and bound water further illustrates the role of the combined use of CCK and LS in hydration. After 28 days of hydration, the PGC blend displayed the highest content of C-(A)-S-H gel, as evidenced by its elevated bound water content and reduced portlandite content. This result indicates that the depletion of portlandite restricts further pozzolanic reactions within the PGC system. In contrast, the PGLC blend exhibited superior mechanical properties compared to PGC, attributable to the filling effect of LS and the refinement of pore structure by carboaluminates. For the PGLC samples, enhanced pozzolanic reactions were observed with increasing incorporation of CCK. Although PGLC samples maintained a similar bound water content after 28 days of hydration, the formation of carboaluminates appears to significantly contribute to the improvement in mechanical properties.

### 3.3. Effect of CCK and LS on the Cement Pore Structure

**Figure 10:**
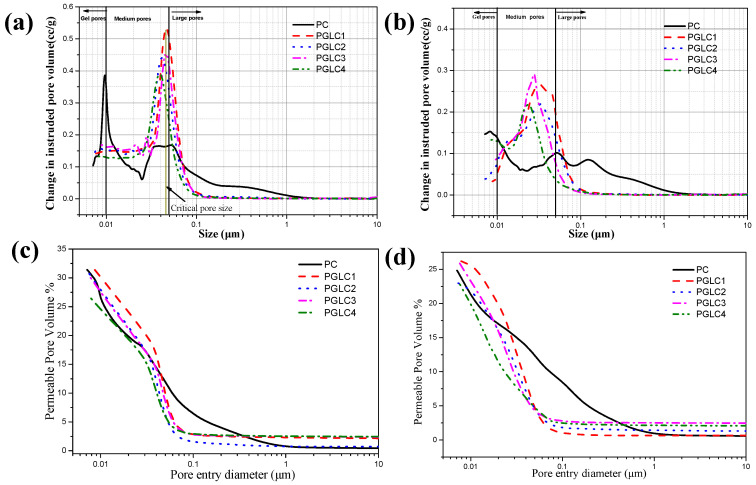
Pore size distribution of blends after (**a**) 7 d and (**b**) 28 d of hydration; porosity distribution of blends after (**c**) 7 d and (**d**) 28 d of hydration.

Based on the relationship between pore size and the overall performance of the cement system, the pore structure in the hydrated samples can be categorized into gel pores (<0.01 μm), medium pores (0.01–0.5 μm), and large pores (>0.5 μm). Figure 10 illustrates the pore size distribution Figure 10a,b and porosity distribution Figure 10c,d of the samples after 7 days and 28 days of hydration. After 7 days of hydration, the incorporation of CCK and LS slightly refined the pore distribution of the system. The samples exhibited similar critical pore sizes, which corresponded to the strength and X-ray diffraction results. The addition of CCK and LS converted harmful large pores into less harmful medium pores during the hydration period. Notably, the differential volume curves for the ternary system shifted clearly to the left; the tendency of the shift is related to the mass ratio of CCK to LS. Furthermore, the critical pore size decreased at 28 days, indicating that this refinement of the pore structure contributes significantly to the improvement of system strength.

At 28 days, the DoH of the clinker is comparable to the ternary system. When considered alongside hydration and strength results, the presence of additional hydration products is identified as a key factor influencing pore refinement and strength development at later stages of hydration. As the mass ratio of CCK to LS increases, the generation of additional hydration products facilitates improved pore structure evolution. This suggests that carboaluminate plays a significant role in refining the pore structure of cement during the later hydration period. Compared to PGLC3, PGLC4 demonstrates a slight improvement in both pore structure and mechanical properties. To mitigate the CO_2_ footprint of the cement industry, a replacement ratio of 2:1 for CCK and LS is recommended as an effective option within this system.

## 4. Conclusions

This paper investigates the effects of varying mass ratios of CCK and LS with a 45% cement substitution. Based on the results regarding hydration, mechanical properties, and pore structure, the following conclusions can be drawn:

(1) The dilution effect of CCK and LS dominate the strength development at early hydration age, as all ternary blends represent a similar compressive strength of cement at 3 days; however, the synergistic effect of CCK and LS significantly promotes further strength development at a late hydration age. The highest compressive strength reached 47.2 MPa at 28 days. An optimal mass ratio of CCK to LS exists, wherein a ratio of 2:1 enables the blends to achieve comparable strength development.
(2) CCK and LS notably influence the early hydration of cement within the first day, as evidenced by the inverse relationship between heat release and CCK content. Additionally, both CCK and LS promote a more pronounced exothermic process during hydration; all PGLC blends show similar total heat release results, and PGLC 4 represents 86.2% of total heat release as compared to reference PC after 80 h of hydration.
(3) Both CCK and LS enhance the DoH of clinker throughout the hydration process, stimulating the formation of additional C-(A)-S-H and carboaluminates by consuming portlandite. The content of MC in the system is higher than 10 wt% after 56 days of hydration, and the content of CCK in raw materials only has slight impact on the content of MC at late hydration age. The additional hydration products optimized the pore structure evolution at a late hydration age; the reduction of harmful pores favors the further strength development of the system at late hydration.
(4) This work establishes that the combination of CCK and LS is an ideal design for SCMs. However, it is important to consider that the incorporation of CCK increases the CO_2_ footprint of these blends, necessitating an optimization of the mass ratio of CCK to LS. In terms of balancing CO_2_ emissions and blend performance, a CCK–to–LS ratio of 2:1 exhibits comparable performance under the conditions explored in this study. This work provides a new insight into the application of LC^3^ systems and reduces the CO_2_ footprint in the cement industry.

## Figures and Tables

**Table 1:** Chemical composition of raw materials and the XRD–Rietveld refinement of clinker.

**Chemical Composition (%)**		**SiO_2_**	**Al_2_O_3_**	**Fe_2_O_3_**	**TiO_2_**	**CaO**	**MgO**	**SO_3_**	**K_2_O**	**Na_2_O**	**TOL**
	Clinker	21.29	5.66	3.78	0.29	65.99	1.16	0.37	0.50	0.32	0
	Gypsum	6.13	1.64	0.44	0.09	31.98	1.21	36.35	0.35	0.12	21.07
	Limestone	0.85	0.32	0.31	0.027	54.19	0.89	0.035	0.12	0.011	43.06
	CCK-raw	44.79	23.32	7.80	1.96	2.13	1.07	0.32	1.14	0.16	18.78
	CCK-750	50.22	28.83	9.03	2.52	2.26	1.24	0.39	1.33	0	3.33
Phase composition (%)		C_3_S	C_2_S	C_3_A	C_4_AF	CaO	CaSO_4_		Blaine specific surface		
	Clinker	66.03	9.26	12.11	8.62	1.95	0.06		314 m^2^/kg		

**Table 2:** Mixture design.

	Clinker (%)	Gypsum (%)	CCK (%)	LS (%)
PC	95	5	-	-
PLC	52.25	2.75	-	45
PGC	52.25	2.75	45	-
PGLC1	52.25	2.75	15	30
PGLC2	52.25	2.75	22.5	22.5
PGLC3	52.25	2.75	30	15
PGLC4	52.25	2.75	35	10

## Data Availability

The original contributions presented in the study are included in the article; further inquiries can be directed to the corresponding authors.
